# Structural, Optical and Mechanical Properties of Nanocrystalline Molybdenum Thin Films Deposited under Variable Substrate Temperature

**DOI:** 10.3390/ma15030754

**Published:** 2022-01-19

**Authors:** Nanthakishore Makeswaran, Cristian Orozco, Anil K. Battu, Eva Deemer, C. V. Ramana

**Affiliations:** 1 Centre for Advanced Materials Research (CMR), University of Texas at El Paso, 500 W University Ave, El Paso, TX 79968, USA; nmakeswaran@miners.utep.edu (N.M.); corozco7@miners.utep.edu (C.O.); anilkrishna.battu@gmail.com (A.K.B.); eva.deemer@gmail.com (E.D.); 2 White Sands Missile Range (WSMR), White Sands, NM 88002, USA; 3 Environmental Molecular Sciences Laboratory (EMSL), Pacific Northwest National Laboratory (PNNL), Richland, WA 99352, USA; 4 Department of Chemistry and Biochemistry, University of Texas at El Paso, 500 W University Ave, El Paso, TX 79968, USA

**Keywords:** molybdenum, thin films, morphology, mechanical properties, optical constants

## Abstract

Molybdenum (Mo), which is one among the refractory metals, is a promising material with a wide variety of technological applications in microelectronics, optoelectronics, and energy conversion and storage. However, understanding the structure–property correlation and optimization at the nanoscale dimension is quite important to meet the requirements of the emerging nanoelectronics and nanophotonics. In this context, we focused our efforts to derive a comprehensive understanding of the nanoscale structure, phase, and electronic properties of nanocrystalline Mo films with variable microstructure and grain size. Molybdenum films were deposited under varying temperature (25–500 °C), which resulted in Mo films with variable grain size of 9–22 nm. The grazing incidence X-ray diffraction analyses indicate the (110) preferred growth behavior the Mo films, though there is a marked decrease in hardness and elastic modulus values. In particular, there is a sizable difference in maximum and minimum elastic modulus values; the elastic modulus decreased from ~460 to 260–280 GPa with increasing substrate temperature from 25–500 °C. The plasticity index and wear resistance index values show a dramatic change with substrate temperature and grain size. Additionally, the optical properties of the nanocrystalline Mo films evaluated by spectroscopic ellipsometry indicate a marked dependence on the growth temperature and grain size. This dependence on grain size variation was particularly notable for the refractive index where Mo films with lower grain size fell in a range between ~2.75–3.75 across the measured wavelength as opposed to the range of 1.5–2.5 for samples deposited at temperatures of 400–500 °C, where the grain size is relatively higher. The conductive atomic force microscopy (AFM) studies indicate a direct correlation with grain size variation and grain versus grain boundary conduction; the trend noted was improved electrical conductivity of the Mo films in correlation with increasing grain size. The combined ellipsometry and conductive AFM studies allowed us to optimize the structure–property correlation in nanocrystalline Mo films for application in electronics and optoelectronics.

## 1. Introduction

Realizing the full potential of electronic materials for integration into technological applications lies with the manipulation of their structure and properties at the nanoscale dimension. Understanding the structure–property relationship as a function of processing conditions is a critical aspect of cutting-edge research in the application of electronic and optical materials for a wide array of applications such as integrated sensors, thin film solar cells, optical filters, and microelectromechanical systems (MEMS) [1,2,3,4,5]. Especially, a fundamental understanding of the crystallography, microstructure, surface/interface chemistry, defect chemistry and their evolution as a function of synthetic conditions is critically important and allows the fine tuning of material properties for viable electronic device applications and to explore new applications [6,7,8,9,10,11].

Molybdenum (Mo), which is a refractory metal, is a promising material for a wide variety of applications in the current and emerging micro/nano-electronics. Mo thin films find widespread applications in electronic and optoelectronic devices, which include integrated circuits, transistors, transition-edge sensors (for X-ray reflectometry), interconnects, diffusion barriers, MEMS, thin film solar cells and radiation detectors [12,13,14,15,16,17,18,19]. The extensive usage of Mo in electronic device applications is made possible by virtue of its remarkable properties, namely, high melting point, excellent chemical stability and mechanical properties, and high electrical conductivity. Furthermore, the refractory nature of Mo along with its high melting point (2623 °C) greatly expands the commercial and scientific uses of the material for operation under extreme environment conditions [20,21,22,23,24,25,26,27,28,29,30,31,32]. From an electronic material perspective, the most important to mention is its emergence as the predominant choice as the electrically conducting back contact electrode for thin film solar cells. The success and high efficiency of Cu(In)Se_2_ (CIS)- or Cu(In,Ga)Se_2_ (CIGS)-based polycrystalline thin film solar cells have been partly attributed to the role played by the Mo back contact layer. Mo fulfills the ideal requirements for an effective back contact, notably chemical and mechanical compatibility with the other deposition processes, high conductivity, low contact resistance with the CIS/CIGS layer, and commensurate thermal expansion coefficient. In applications such as back contacts for CIS and CIGS solar cells, Mo shines not only due to its electrical properties but also for the inertness and mechanical durability it displays during absorber film growth [25,27,33]. Maintaining a metallic state, i.e., without oxidation, and retaining the structural quality and desirable electrical properties of Mo at the high processing temperatures, a common aspect for many energy and electronic industries is now the main challenge in expanding the role of Mo in the commercial and scientific world [27,34,35,36].

A fundamental, deeper understanding of the nano-structure and size effects on the electrical, mechanical and optical properties of nanocrystalline Mo films is very important for all the aforementioned applications. Molybdenum thin films for electronic device applications are commonly produced by physical vapor deposition methods [20,21,29,30,37,38,39,40]. However, it has been demonstrated that the radio-frequency (RF) magnetron sputtering represents an alternative approach to deposit high-quality Mo films for electronic and optoelectronic applications [4,40,41]. RF sputtering has been shown to deposit high quality Mo films, though there have been cases of desirable electrical properties being counterbalanced by poor adhesion, implying optimizing the processing conditions is the key to produce high-quality layers with desirable properties. Sputtering pressure has been shown to vary the resistivity and adhesion of RF and DC magnetron-sputtered Mo thin films [20,21,29,30,37,38,39,40,41]. Jubault et al. demonstrated that the sputtering pressure influences the adhesion of Mo layers [4]. Reasonably good adhesion of Mo could be obtained within a much wider range of sputtering pressures; however, electrical properties of Mo were shown to be less sensitive [4]. Zoppi et al. reported the growth and optimization of electrical of properties of Mo films [25]. They showed that Mo films with a low resistivity (21.3 μΩ-cm) can be obtained for deposition at room temperature through the manipulation of the RF process parameters [25]. It has also been shown that a low resistivity of 22.8 μΩ-cm for Mo films by RF sputtering can be achieved using a slightly higher deposition temperature (200 °C). However, further increasing deposition temperatures caused degradation in the overall properties [40]. Recently, Dai et al. demonstrated the superior characteristics of Mo films deposited by RF sputtering. In their work, Mo films were deposited at an elevated deposition temperature (400 °C) with varying sputtering power and sputtering gas pressures [41]. In these experiments, lower pressure and higher power of RF sputtering result in Mo films with lower resistivity due to increased kinetic energy of sputtered particles, this in turn improved the crystallinity and compactness of the films [41]. However, while desirable electrical properties can be obtained, adhesion properties of the Mo films were very poor [41]. Thus, it is obvious that the optimization of conditions by means of a comprehensive understanding of the structure and properties as a function of processing conditions is essential to utilize Mo films in the desired technological applications. Most importantly, understanding the linkage between microstructure and electronic properties in nanocrystalline Mo films is critical. Therefore, in the present work, a combined ellipsometry and conductive atomic force microscopy (c-AFM) study was made to understand the effect of nanostructured grains and grain boundaries on the electrical and optical properties of Mo films. It is well known that the physical properties of the nanoscale materials including hardness, elastic modulus, toughness and durability play a critical role in long-term viability in electronics and energy applications and are closely controlled through defects, grain boundaries, and interfaces [15,16,17,18,19,20]. For metals and alloys, grain boundaries represent a comparatively high-volume fraction of a material system and will strongly influence its properties [12,13,14]. In the present work, an attempt is made to fabricate Mo films under variable substrate temperatures so as to produce materials with variable grain size at the nanoscale dimensions and understand their structural, mechanical, electrical and optical properties.

## 2. Experimental Details

### 2.1. Fabrication

The molybdenum (Mo) thin films were deposited onto Si (100) wafers through the use of radio-frequency sputtering. We adopted the previously established procedures and methods to fabricate Mo films and the details of the deposition chamber, an Excel Model DCSS-12, were reported elsewhere [6,42]. The wafers were cleaned with ethyl alcohol and dried with nitrogen before placement in the vacuum chamber. The chamber was then evacuated to a base pressure of ∼10^−7^ Torr. A 2-inch diameter Mo target of 99.95% purity (Plasmaterials, Inc., Livermore, CA, USA) was used in conjunction a 2-inch diameter sputter gun placed 7 cm from the substrate. A sputtering power of 40 W was initially applied to the target while argon (40 sccm) was introduced to the chamber to ignite the plasma, with the gas flow being controlled with an MKS mass flow meter(Andover, MA, USA). Once ignition had been achieved, the power was increased to 100 W for actual depositions while final deposition pressure was tuned to 5 mTorr. Pre-sputtering was done for 15 min with a shutter closed above the gun. During deposition, the temperature of the substrate was varied from room temperature (~25 °C) to 500 °C. The deposition time for each substrate was kept constant at 30 min with constant rotation of the substrates at 3–4 rpm implemented to ensure lateral isotropy. While several iterations were made by producing at least a set of 4 samples under each and every deposition temperature, one set of samples was prepared for each deposition temperature before proceeding to characterization. Thus, we believe that the results presented and discussed in this paper present an overall picture of the effect of deposition temperature and the grain size. The average measured thickness of the deposited Mo films, as determined via ex situ spectroscopic ellipsometry, is 120 nm, and while there is an initial increase in film thickness with applied power to the sputtering gun, which is to be expected, there is a sharp decrease between the 300 and 400 °C samples with a slight increase again with the increase to 500 °C.

### 2.2. Characterization

#### 2.2.1. Ellipsometry

Spectroscopic ellipsometry (SE) measurements were employed to determine the electronic parameters and microstructure characteristics. The ellipsometric angles Ψ and Δ were determined over the wavelength range of 300–1800 nm using a J.A. Woolam alpha-SE Ellipsometer (Lincoln, NE, USA). All the measurements were made ex situ and at room temperature. The incident angles were selected between 65 and 75 degrees and a 10 s/angle rate was set for data acquisition. Fitting of the acquired data curves was accomplished with the CompleteEASE acquisition software [42] using a Drude–Lorentz model based on the following equation:

(1) $$\varepsilon \left(\omega \right)=\varepsilon_{\infty }-\frac{\omega_{pu}^{2}}{\omega^{2}-i\Gamma_{D}\omega }+\sum_{j=1}^{2}\frac{f_{j}\omega_{0j}^{2}}{\omega_{0j}^{2}-\omega^{2}+i\gamma_{j}\omega }$$

where the Drude term is determined by the unscreened plasma energy ℏω*_pu_* and the damping factor Γ_*D*_. The lorentz oscillators are determined at energy position ℏω_0*j*_, with strength *f_j_* and damping factor *γ_j_* [43,44].

#### 2.2.2. Grazing Incidence X-ray Diffraction (GIXRD)

**Figure 1:**
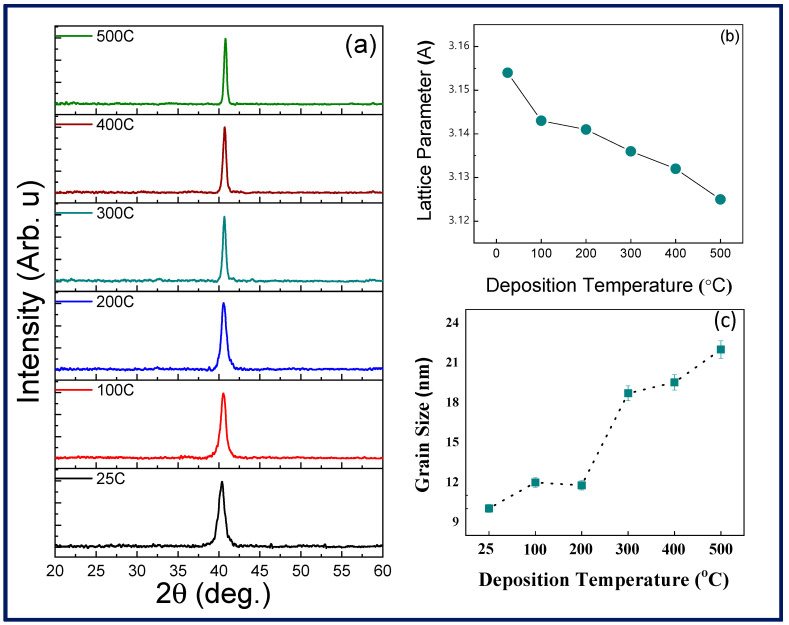
(**a**) θ/2θ X-ray diffraction patterns of Mo films deposited at different temperatures. The (110) peak is the only peak observed; the peak intensity increases with increasing deposition temperature. The broader peak for Mo films deposited at 25–200 °C indicates the very small crystallite size. (**b**) The lattice parameter variation with deposition temperature. The lattice parameter increase at lower temperatures is primarily due to nanoscale effects. (**c**) The variation of average crystallite size with deposition temperature. It is evident that the average crystallite size increases with increasing deposition temperature.

Structural characterization of the samples was performed using Grazing Incidence X-ray Diffraction at room temperature utilizing a Bruker D8 advance system (Billerica, MA, USA). (Cu-Kα radiation, and λ = 1.54 Å). To focus on the surface layer of the film, a grazing incident angle of 1 degree was selected for the incoming X-rays. The detector scanned between 10–67° with a speed of 0.5 s/step. The crystallite size and lattice parameter of the samples were calculated using standard XRD procedures [45]. The average crystallite size in nm (Figure 1c) was determined using the Scherrer equation [45]:

(2) $$d=\frac{0.9\lambda }{\beta \cos\theta }$$

where λ represents the X-ray wavelength, β is the half intensity peak width, and d is the average crystallite size.

#### 2.2.3. Atomic Force Microscopy

To determine defects in conductive Mo films in terms of local resistance or local transport, scanning spreading resistance measurements were performed. A conductive probe comes into contact with the sample surface and a bias voltage is applied to the probe and the current flow is measured as a function of the probe simultaneously with surface topography. Scanning Spreading Resistances were scanned at a frequency of 0.6 Hz using golden silicon probes (NT-MDT CSG10/Pt, tip curvature radius of 35 nm) with a force constant 0.1 N/m. A bias voltage of 0.010 V was applied to the sample through the tip. Surface roughness, *R_a_*, is calculated using [46]:

(3) $$R_{a}=\frac{1}{L}\overset{L}{\underset{0}{\int }}\left|Z\left(x\right)\right|dx$$

where *L* is the evaluation length, and *Z*(*x*) acts as a function of the height *Z* and position *x* of the sample. This surface roughness is the height variation along a single line profile or a set of parallel profiles.

#### 2.2.4. Mechanical Properties

The mechanical characteristics, namely hardness (H) and elastic modulus (E_r_), were determined for the deposited Mo films. All the mechanical testing measurements were made using a Hysitron T1750 Tribo nanoindentor (Bruker, Billerica, MA, USA). A triangular pyramid Berkovich diamond indenter with a normal angle of 65.3° was used with an effective size of the apex of roughly 100 nm. The method developed by Oliver and Phar [47] was utilized to calculate hardness and elastic modulus with the use of the unloading curve slope. Using the stiffness (*S*) of the film from the slope of the unloading curve and maximum load (*P_max_*), E_r_ and H values were determined from the relations:

(4) $$E_{r}=\frac{\sqrt{\pi }}{2}\;\frac{S}{\sqrt{A}};\;H=\frac{P_{max}}{A}$$

where *A* is defined as the area of contact at peak load. Several indentations were performed on each and every Mo sample while the estimated statistical average of H and E_r_ values were reported.

#### 2.2.5. Scratch Testing

Scratch testing measurements were used to understand the Mo film adhesion to the substrate and were analyzed by a nano-scratch test (Hysitron T1750 Tribo nanoindentor). For nanostructured samples, film or coating, the adhesion to the substrate is one of the paramount characteristics related the coating quality and life endurance. This test was performed at room temperature under the load increments from 0–8000 μN. The same standard triangular pyramid Berkovich diamond indenter tip has been used. The length of the scratch was 16 μm, and the scratch speed was 0.18 μm/s.

## 3. Results and Discussion

### 3.1. X-ray Diffraction—Crystal Structure and Phase

The crystal structure data and analyses of Mo films are shown in Figure 1. The XRD patterns for the Mo samples (Figure 1a) indicate that all the samples are crystalline. The XRD peak appearance at ~40.5° indicates successful deposition of nanocrystalline Mo films. Also, the evolution of the XRD peak indicates the nc-Mo preferred (110) growth. It should be noted that with increased temperature there is a shifting of the peaks as well as sharper peak formation. This general trend with an increase in sharpness of observed peaks indicates an increase in crystallinity and the average crystal size with increased deposition temperature. The lattice parameter variation is shown in Figure 1b. The lattice parameter, which is calculated from XRD data, indicates more or less a linear trend in the reduction. Also, it is noted that this trend in lattice parameter variation is largely inverse to the average crystallite size shown in Figure 1c.

It is evident that there exists a direct correlation between the average crystallite size and the deposition temperature of Mo films.

### 3.2. Surface Morphology and Electrical Conduction—Atomic Force Microscopy

**Figure 2:**
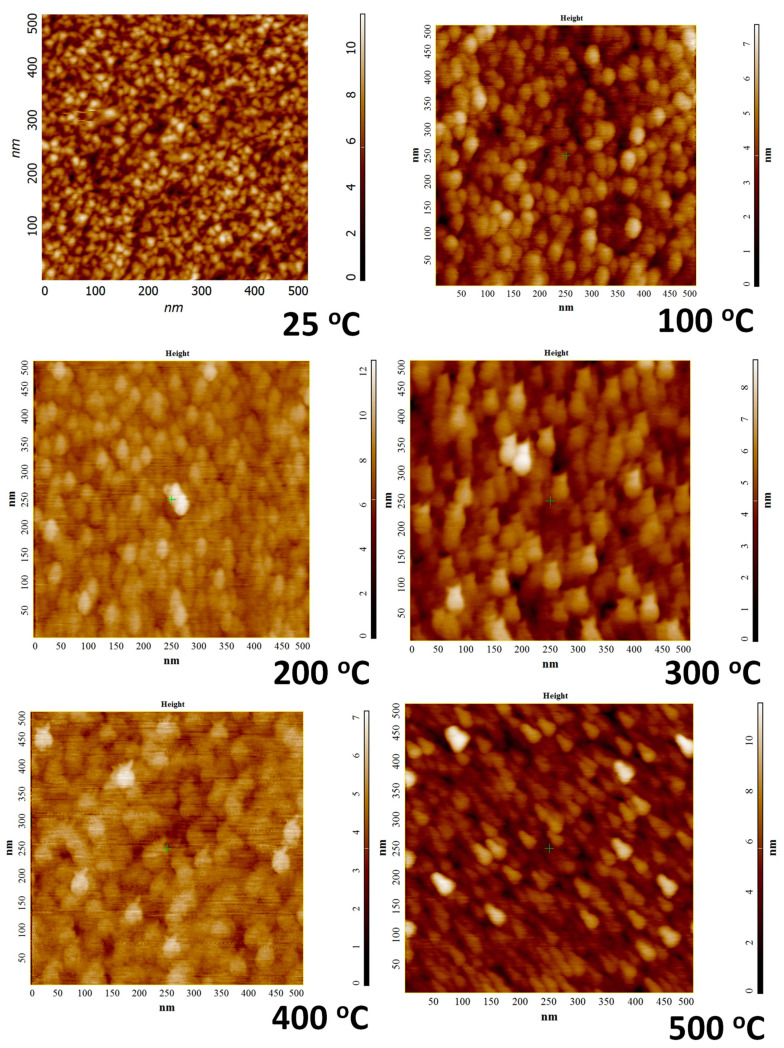
AFM topographic surface images of Mo films deposited under variable deposition temperature. The morphology evolution with increasing deposition temperature is evident. Also, the granular morphology with relatively smaller grain size formation at lower deposition temperatures can be seen in the AFM images.

**Figure 3:**
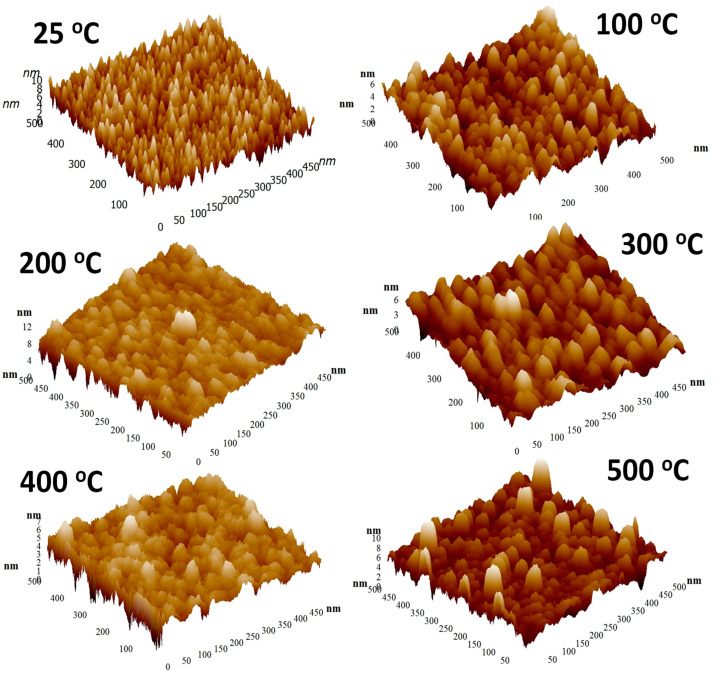
The corresponding three-dimensional surface AFM images of Mo films deposited at various temperatures. It is evident from these images that the grain size and surface roughness evolution in Mo films is dependent on the deposition temperature.

**Figure 4:**
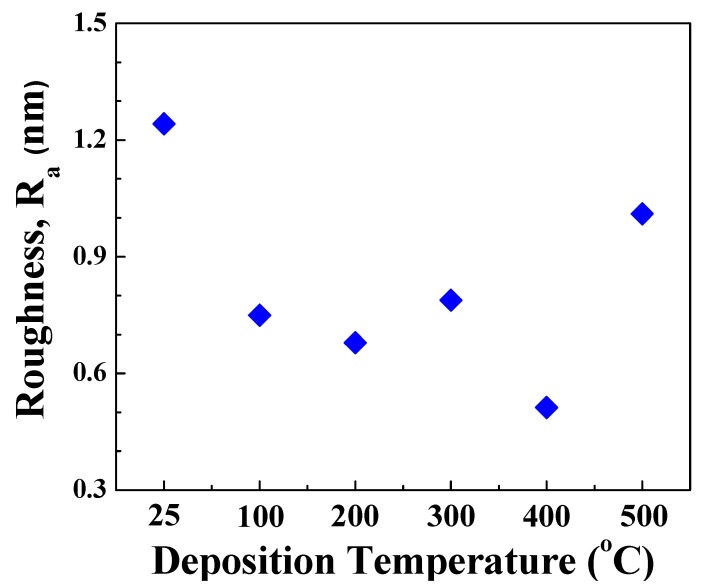
Variation of surface roughness of Mo films with deposition temperature.

The surface morphology probed by AFM indicates that the deposition temperature influences the morphology evolution of the Mo films. The AFM data of the Mo films deposited under variable substrate temperature are shown in Figure 2 and Figure 3. The topographic images of the morphology is shown in Figure 2 while the three-dimensional surface plots are shown in Figure 3. It is evident that all the Mo films exhibit the granular morphology. However, the grain size is relatively small and increases with increasing temperature. This correlates well with the general trend observed in the XRD measurements with an increase in sharpness of observed peaks indicating an increase in crystallinity and the average size with increased deposition temperature. Also, at the very beginning, i.e., for deposition temperatures 25–200 °C, the morphology of the Mo films is characterized by the presence of nearly spherical grains. It is evident that such characteristic morphology seen at lower temperatures is not present for Mo films deposited at temperatures > 200 °C. This can be attributed to the grain growth and grain size increase with increasing deposition temperature. Also, such growth behavior may be anisotropic, leading to the observed variations in the surface morphology as a function of deposition temperature. To obtain quantitative information on the surface morphology, various single numerical parameters, Ra and Rq, are useful mainly for classifying surfaces of the same type that are produced by the same method [48]. The average roughness (Ra) is well-correlated with the Root Mean Square (Rq). The surface roughness has a decreasing trend, non-monotonically, however, with increasing temperature. The deposition temperature has a dramatic effect on surface roughness, as shown in Figure 4.

**Figure 5:**
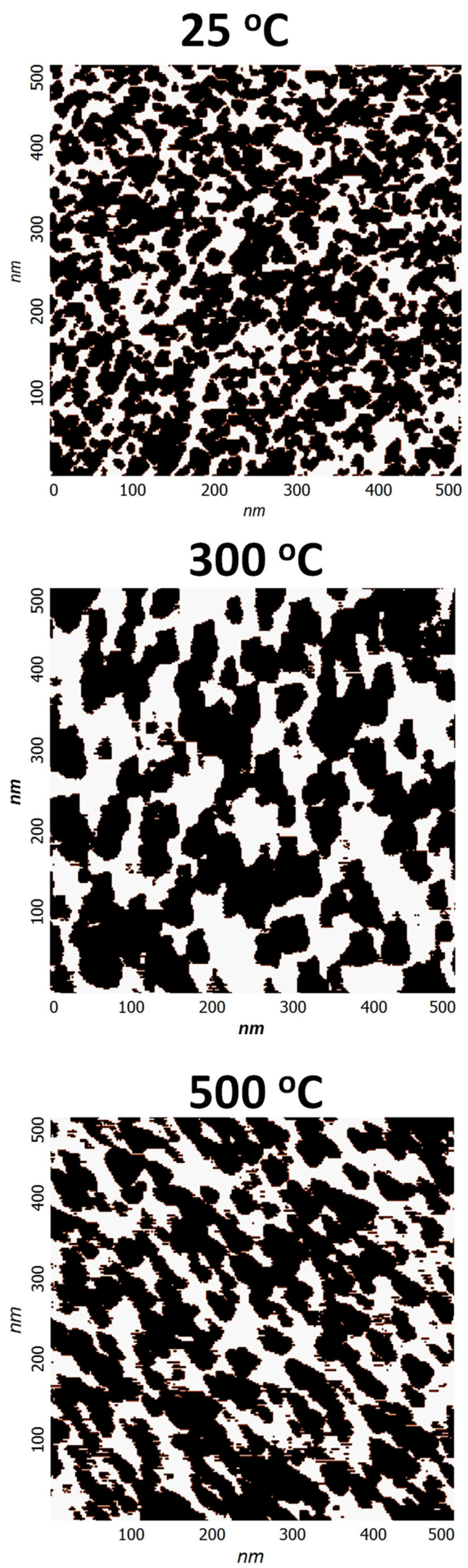
Grain analysis for Mo films deposited at different temperatures. As indicated in the images, as the temperature increases, the grain size increases gradually while intra-grain voids become less and less visible.

In addition to surface morphology and surface roughness, AFM phase plots were used to understand the grain structure and analysis as a function of deposition temperature. The deposition temperature affects the gas-phase collisions and increased temperature increases the kinetic energy of the sputtered Mo species leading to the dense morphology of the Mo film with less intra-grain voids porous grains. This is indeed observed in the phase-grain AFM imaging analysis shown in Figure 5. Also, the grain size variation, based on the statistical data analysis of the diameter of the grains detected in the specific surface area of the AFM image, is 9–20 nm for a variation in substrate temperature from 25–500 °C. However, while the grain size values reasonably agree with those obtained from XRD data for a temperature up to 300 °C, the size determined in AFM tends to be smaller than that of XRD. However, corroborating with XRD results, as the temperature increases, the grain size increases gradually while intra-grain voids become less and less visible. This characteristic morphology, perhaps, is expected to strongly influence the mechanical and optical properties.

**Figure 6:**
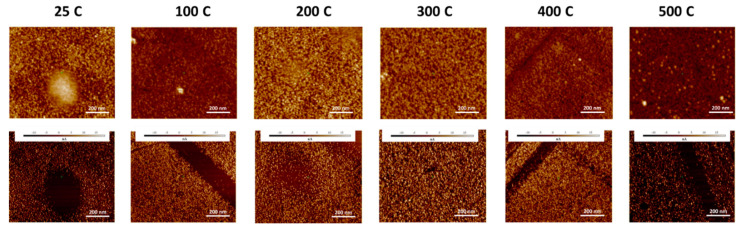
AFM height (**top**) and current (**bottom**) micrographs of Mo films at different pressures.

The electrical properties of Mo films were investigated by Scanning Spreading Resistance during Atomic Force Microscopy. Topological features correlate with current profiles and it can be seen that there are features that translate to decreases in current. In Figure 6, it can be seen that with increasing temperature, the densification and increase in crystallinity to 400 °C can be visually observed, though upon reaching 400 °C there is a decrease in surface roughness. In Figure 6, after 400 °C there seems to be two regimes of larger grains competing with smaller grains instead of a well-distributed average grain size. At higher temperatures, there is higher energy to enhance the sputtered atom surface mobility. This results in the densification of microvoids and correlates to better crystallinity, larger grain size, fewer voids and a lower concentration of impurities, which tends to support the reduction in lattice parameter reported in the XRD findings. Therefore, at higher temperatures, the Mo films have higher conductivity. Zhou et al. also show this increase in crystallinity with temperature and report optimization in Mo film conductivity at 400 °C [49]. Our results confirm this observation and demonstrate that temperatures above 400 °C form crystallites of two different sizes and a corresponding decrease in current density.

### 3.3. Optical Properties—Ellipsometry

**Figure 7:**
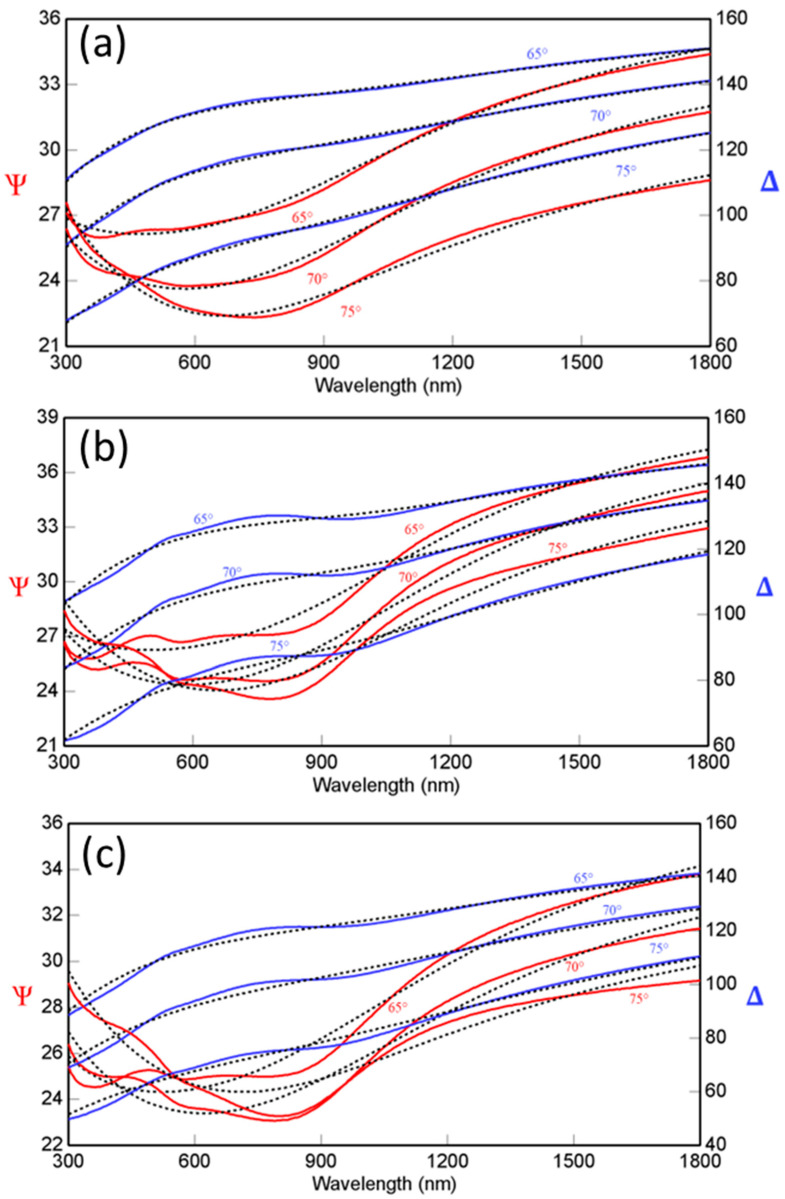
The spectral dependencies of the SE functions, Ψ and Δ, measured for nanocrystalline Mo samples. The experimental and simulated curves (as indicated by the solid and dashed lines respectively) for Δ and ψ are shown. The data shown are for Mo samples deposited at—(**a**) 25 °C, (**b**) 300 °C, and (**c**) 500 °C. A high degree of agreement between the curves is indicative of accurate modeling and is needed for reliable derivation of optical constants.

The optical constants, namely the index of refraction (*n*) and extinction coefficient (*k*), and their dispersion profiles, which are quite important and interlinked to the film surface/interface quality and characteristics, were revealed by the SE analyses. The spectral dependencies of the SE functions, Ψ and Δ, determined for nanocrystalline Mo samples, are shown in Figure 7. The spectral dependencies of Ψ and Δ were fitted with model, as mentioned under the experimental section, to extract the optical constants, i.e., the refractive index (*n*) and extinction coefficient (*k*), based on the best fit between experimental and simulated spectra. The data as shown in Figure 6 show a reasonable agreement between the experimental and simulation data for the entire range of depositions made.

**Figure 8:**
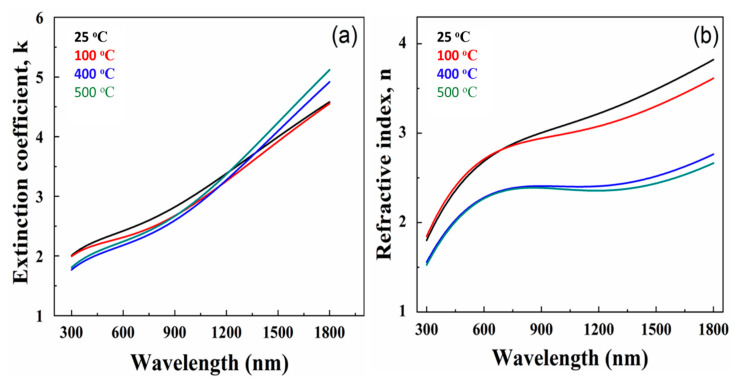
(**a**) Extinction Coefficient (k), and (**b**) Refractive index (n) as a function of wavelength. Data are shown for samples at 25 °C, 100 °C, 400 °C, and 500 °C.

Figure 8 displays the variation in extinction coefficient and refractive index for the set of Mo films. It should be noted that all samples were measured accounting for inherent surface roughness. Two important observations that can be made from the dispersion profiles of optical constants are as follows. The first is that for all the Mo films, both *n* and *k* values increase with increasing wavelength. In fact, the dispersion profiles noted for Mo films are reasonably good with a typical behavior expected for metals, in general, or metal thin films [50,51,52]. The marked dependence of the optical constants on the deposition temperature is the second. Such dependence is more clearly seen in the *n*-values as a function of deposition temperature. The *n*-profiles exhibit a general decreasing trend with increasing deposition temperature, particularly noticeable at higher wavelengths. The observed differences can be accounted for by considering the microstructure and morphology variation of the Mo films with deposition temperature. The average grain size increases with increasing deposition temperature. Therefore, the higher *n*-value trend may be due to smaller grain size. Increasing temperature increases the grain size, which in turn reduces grain boundary scattering, and causes variation in optical properties.

For polycrystalline metal and metal-oxide films, the optical constants are sensitive to the crystal structure, morphology, microstructure, defect structure and chemistry [50,51,52,53,54]. Most importantly, the surface/interface structure, crystal quality, packing density, lattice parameters, and defect structure of the deposited films strongly influence their optical parameters [50,51,52,53,54]. Thus, the observed changes noted for Mo films as a function of deposition temperature can be attributed to the microstructure and grain size variation. Usually, the low-packing density and/or defects can result in relatively low values, which can influence the *n* and *k* values of the metal films [52]. As reported in the literature, sputtered films can contain a number of impurities, which may be incorporated during deposition either from the residual gas or from the sputtering gas. In fact, Walker et al. reported that, for a given thickness of Mo film, the samples deposited at lower pressure exhibit a higher amplitude imaginary part of the dielectric function at high photon energies which is attributed to stronger optical absorption associated with the interband transitions [52]. It was found that the lower void volume fraction packing density of crystallites in the films influences the optical properties of Mo films [52]. Therefore, we believe that the variation in *n* and *k* values noted in this work are primarily due to the grain size variation and structural order in the Mo films.

### 3.4. Mechanical Properties—Nanoindentation and Nanostratch

**Figure 9:**
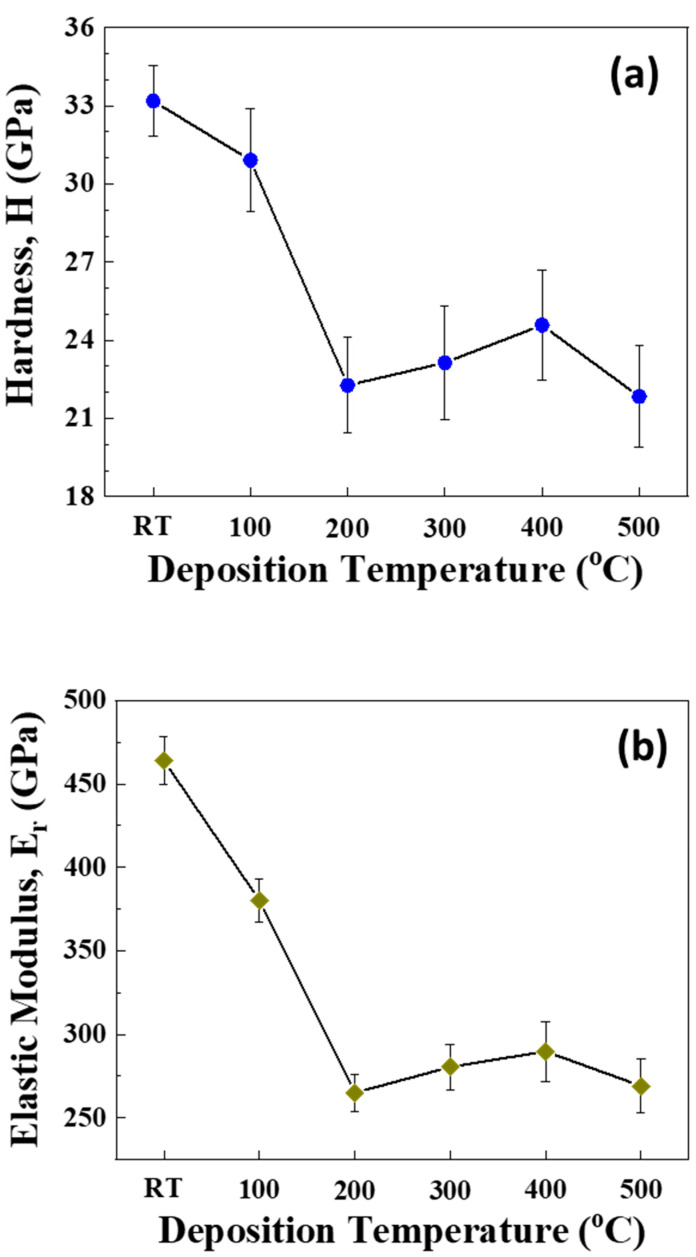
The variation of (**a**) hardness and (**b**) elastic modulus of Mo films with deposition temperature. The higher values of hardness and elastic modulus for Mo films deposited at 25–200 °C are due to the size reduction effect while the decreases observed for Mo films deposited at 200 °C are due to the increase in average crystallite size. Similar trend in both sets of values are indicative of the interconnected nature of these properties.

Indentation testing of the samples yielded a similar trend for both hardness (H) and elastic modulus (E), as shown in Figure 9. It can be seen that both H (Figure 9a) and E (Figure 9b) values decrease continuously with increasing deposition temperature, although the decreasing trend slows down at higher temperatures. This demonstrates an initial increase in fine-structured Mo nanomaterial, while those values tend to decrease with increasing size and formation of more crystalline Mo films. The variation in mechanical characteristics (H and E) for Mo films as a function of deposition temperature can be understood as follows. As reported widely in the literature, the mechanical strength of polycrystalline metals and alloys can be significantly improved by controlling the crystallite size [55,56,57,58,59,60,61]. Smaller size contributes to the enhanced strength. The slip is arrested along the grain boundaries which further concocts a strengthening effect [55,56,57,58,59,60,61]. For metals, the size dependence of the mechanical properties can be explained on the basis of the Hall–Petch relationship which is represented as [59]:

(5) $$\sigma_{y}=\sigma_{0}+kd^{-1/2}$$

where $\sigma_{0}$ is the friction stress resisting the motion of gliding dislocation, and *k* is the Hall–Petch slope, which is associated with a measure of the resistance of the grain boundary to slip transfer. Therefore, the relatively higher values of H and E observed for nanocrystalline Mo films deposited at lower temperature can be attributed to the fine-structured nano-sized crystallites. Increasing the deposition temperature, especially at Ts > 200 °C, increases the size resulting in the observed decrease in H and E values. Enhanced H and E values noted under the d-Ts relationship for Mo films was also noted for other metals, such as Tantalum, Copper, Nickel, and Nickel-Tungsten alloys [61,62,63].

**Figure 10:**
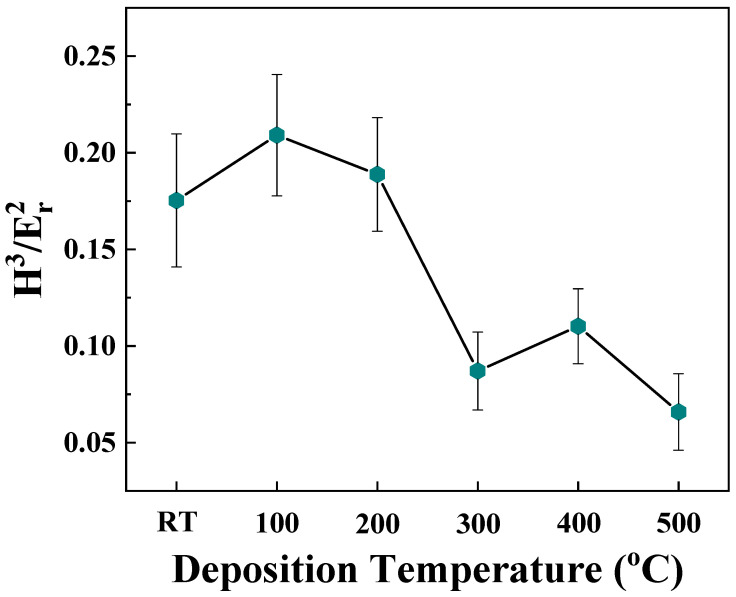
The variation of H^3^/E^2^ ratio, which is called the plasticity index, for Mo films deposited at various temperatures. Note that lower values of average crystallite size and finer microstructure in a temperature range under 300 °C indicates a region of superior coating elastic properties and wear resistance.

The ratio H^3^/E^2^, which is generally referred to as the plasticity index of the material, is a reliable indication of a coating’s resistance to plastic deformation. It can be seen (Figure 10) that the H^3^/E^2^ ration is also dependent on the deposition temperature. The ratio decreases with increasing Ts. H^3^/E^2^ is a measure of a film’s cracking resistance and provides firsthand information on the wear performance. The highest values of H^3^/E^2^ for Mo films deposited at temperatures in the range of 25–200 °C are primarily due to the lower values of average crystallite size and the fine microstructure. Since the plasticity index is largely used to determine the limit of elastic behavior in a material which in this case is the wear resistance of the Mo samples, there seems to be an optimal range of temperatures in regards to coating elastic properties under 300 °C.

**Figure 11:**
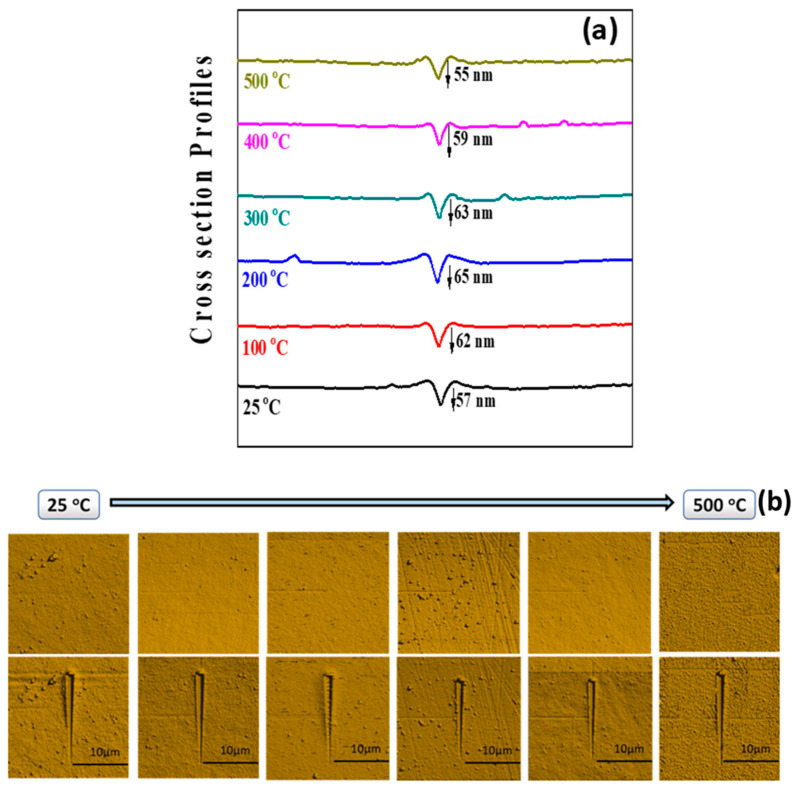
Nanoscratch test results of Mo films. The data shown are—(**a**) cross-section profiles under scratch testing; (**b**) scratch testing images of Mo films.

The scratch test results of Mo films as a function of deposition temperature are shown in Figure 11. The adhesion characteristics of Mo films and the dependence on deposition temperature are evident. The results can be understood from the two considerations. One consideration is cohesive failure which involves deformation in the area surrounding the scratch without damaging the remainder of the film. The second is the adhesive or interfacial failure, which is attributed to the incision of film along the direction of the scratch which further leads to a complete detachment [58] or catastrophic failure [59]. For Mo films it is noted that they are not susceptible to delamination and instead form pile-up deformations as a result of the applied load. (Figure 11b). Also, the depth profiles show the cross-section of the Mo films at the maximum applied force along the scratch. Positive depth indicates the indenter has moved below the initial surface as opposed to negative scratch depth were the indenter is located above the initial sample surface. The depth profile cross-sections indicate that the indenter penetration is relatively less for Mo films deposited at lower temperatures compared to those deposited at higher temperatures. However, the effect is remarkable for Mo films deposited at 100–300 °C compared to those deposited at 400–500 °C. On the other hand, Mo films deposited at room temperature do not offer much resistance for indenter penetration. Thus, the results of the nano-scratch testing and the trend seen matches very well with hardness (Figure 9). Also, no channel cracking/delamination of the Mo films is noted in these nano-scratch tests.

## 4. Conclusions

Nanocrystalline Mo films were deposited using magnetron sputtering at various temperatures ranging from ~25–500 °C. The varying deposition temperature resulted in the *bcc* Mo films with a (110) texturing and with a variable microstructure. The grain size of Mo films was in the range of 9–22 nm, where the increasing deposition temperature increases the size. The mechanical characteristics are highly dependent on the microstructure and grain size as evident in the hardness and elastic modulus values of Mo films. The plasticity index and wear resistance index values show a dramatic change with temperature and grain size. Corroborating with these microstructure and mechanical characteristics, the optical constants of Mo films also indicate the dependence on the grain size. The refractive index and extinction coefficient profiles clearly indicate that the Mo films with lower grain size exhibit better properties. The conductive atomic force microscopy (AFM) studies indicate a direct correlation with grain size variation and grain versus grain boundary conduction.

## Data Availability

The data necessary to substantiate the claims made are available within the manuscript while any claims made based on the literature were given proper citations to the source of information. Specific data or other information can be available with a reasonable request to the first author of the paper.
